# Infusion of bone marrow derived multipotent mesenchymal stromal cells for the treatment of steroid-refractory acute graft-versus-host disease: a multicenter prospective study

**DOI:** 10.18632/oncotarget.25020

**Published:** 2018-04-17

**Authors:** Sophie Servais, Frédéric Baron, Chantal Lechanteur, Laurence Seidel, Dominik Selleslag, Johan Maertens, Etienne Baudoux, Pierre Zachee, Michel Van Gelder, Lucien Noens, Tessa Kerre, Philippe Lewalle, Wilfried Schroyens, Aurélie Ory, Yves Beguin

**Affiliations:** ^1^ Department of Hematology, CHU of Liège, 4000 Liège, Belgium; ^2^ Laboratory of Cell and Gene Therapy, CHU of Liège, 4000 Liège, Belgium;; ^3^ Department of biostatistics, SIMÉ, CHU of Liège, 4000 Liège, Belgium; ^4^ Department of Hematology, AZ Sint-Jan, 8000 Brugge, Belgium; ^5^ Department of Hematology, AZ Gasthuisberg, 3000 Leuven, Belgium; ^6^ Department of Hematology, ZNA Stuivenberg, 2060 Antwerp, Belgium; ^7^ Department of Internal Medicine, Hematology Division, Maastricht University Medical Center, 6202 AZ Maastricht, The Nertherlands; ^8^ Department of Hematology, UZ Gent, 9000 Ghent, Belgium; ^9^ Department of Hematology, Institut Jules-Bordet, 1000 Brussels, Belgium; ^10^ Department of Hematology, Antwerp University Hospital, 2650 Edegem and University of Antwerp, 2610 Antwerp, Belgium; ^11^ Clinical Research Associate of the Belgian Hematology Society, CHU Sart-Tilman, 4000 Liège, Belgium

**Keywords:** allogeneic hematopoietic cell transplantation, corticosteroid-refractory acute graft-versus-host disease, multipotent mesenchymal stromal cells

## Abstract

The prognosis of steroid-refractory acute graft-versus-host disease (aGVHD) remains poor and better treatments are urgently needed. Multipotent mesenchymal stromal cell (MSC)-based therapy emerged as a promising approach but response rates were highly variable across studies. We conducted a multicenter prospective study assessing the efficacy of 1–2 infusion(s) of cryopreserved, third-party donor bone marrow-derived MSCs for treating grade II-IV steroid-refractory or -dependent aGVHD in a series of 33 patients. MSCs were produced centrally and distributed to 8 hospitals throughout Belgium to be infused in 2 consecutive cohorts of patients receiving 1–2 or 3–4 × 10^6^ MSCs/kg per dose, respectively. All patients received MSCs as the first rescue therapy after corticosteroids, with the exception for one patient who received prior treatment with mycophenolate mofetil (that was still ongoing by the time of MSC therapy). In these conditions, MSC therapy resulted in at least a partial response in 13 patients (40.6%) at day 30 and in 15 patients (46%) within 90 days after first MSC infusion. The corresponding complete response rates were 21.6% (7 patients) and 30% (10 patients), respectively. Only 5 patients achieved a sustained complete response, lasting for at least 1 month. The 1-year overall survival was 18.2% (95% CI: 8.82–37.5%). Higher response and survival rates were observed among patients receiving 3–4 × 10^6^ MSCs/kg for first infusion, as compared with patients receiving 1–2 × 106 MSCs/ kg. Response and survival with MSC therapy for SR/SD-aGVHD remains to be optimized.

## INTRODUCTION

Allogeneic hematopoietic cell transplantation (alloHCT) offers potential curative treatment for a number of hematological malignancies [1]. However, its outcome is compromised by the occurrence of acute graft-versus-host disease (aGVHD), a systemic syndrome in which donor immune cells attack tissues (mainly skin, gut and liver) of the immunocompromised host [2, 3]. It is estimated that 30–60% of transplanted patients develop clinically significant grade II-IV aGVHD after alloHCT [4]. Standard first-line treatment is based on high-dose systemic corticosteroids [5]. However, aGVHD fails to respond to steroids or rapidly recurs during steroid tapering in approximately 30–50% of patients [6, 7]. A number of immunosuppressive agents have been tested for controlling steroid-refractory or steroid-dependent aGVHD (SR/SD-aGVHD), but usually with limited success [6–8]. Patients with SR/SD-aGVHD experience high non-relapse mortality, up to 60-85% at 2 years, partly due to aGVHD by itself but also to cumulative toxicity and susceptibility for infections incurred with additional immunosuppressive therapy [8–10]. Therefore, the development of better strategies to control SR/SD-aGVHD remains crucial. Several new drugs are under investigation, with some of them showing promising results, such as ruxolitinib [11]. Among recent approaches, multipotent mesenchymal stromal cell (MSC)-based therapy has also attracted great interest.

MSCs are non-hematopoietic progenitor cells that can be isolated and expanded from bone marrow (BM) and other connective tissues, and that can differentiate into multiple cell lineages

of mesenchymal origin [12]. Over the last decade, evidence accumulated that MSCs are also endowed with broad anti-inflammatory and immunomodulatory properties *in vitro*, influencing both T, B and innate immune cells [13–15]. Moreover, by expressing low or absent levels of human leukocyte antigen (HLA) class I and class II antigens under normal conditions, they can be transferred across HLA barriers, from third-party donors. These properties made them attractive candidates to explore in the treatment of aGVHD after alloHCT.

Over the last decade, numerous pilot phase I-II studies have explored the use of MSC infusion for SR/SD-aGVHD [16–25]. Although most of them suggested that this approach was safe and potentially effective, response rates were highly variable across studies and the durability of the response as well as the impact on survival were not systematically documented. Preliminary results of the sole yet completed randomized phase III placebo-controlled trial with an industrial MSC product (Prochymal^®^) added to the confusion, by reporting no improvement in overall complete and durable response rate with MSC therapy in addition to institutionally selected second line treatment [26]. Therefore, there is still uncertainty regarding the real clinical effectiveness of MSC therapy in SR/SD-aGVHD, particularly in the multicenter setting.

The significant heterogeneity with regard to MSC manufacturing conditions as well as the wide disparity in the degree of characterization of MSC products across previous studies might have contributed to the discrepancy in their results and to the variable MSC efficacy against aGVHD [27, 28]. Specifically, although the International Society for Cellular Therapy (ISCT) has established minimum criteria for defining MSCs, only few studies reported on all of them [27]. Since 2006, we set up a bank of cryopreserved MSC products from BM samples obtained from healthy donors, at the clinical-grade cell production facility of the University of Liège (Laboratory of Cell and Gene Therapy, CHU and University of Liège, Liège, Belgium). MSCs were expanded in fetal bovine serum (FBS)-supplemented medium and early passaged. The whole process, including donor screening, BM collection, mononuclear cell isolation, MSC expansion, harvesting and cryopreservation, as well as release and quality control criteria, was recently published in details [29]. MSCs were compliant with all ISCT criteria.

Using MSC products from this academic bank, we conducted a multicenter prospective study assessing the efficacy of 1-2 MSC infusion (s) for treating grade II-IV SR/SD-aGVHD.

## RESULTS

### Patient characteristics

Forty patients with grades II to IV SR/SD-aGVHD were recruited among 7 Belgian centers and 1 Dutch center between January 2008 and November 2014. Among them, seven patients were retrospectively excluded at the time of the analysis because of deviation from inclusion/ exclusion criteria. Therefore, 33 patients were finally analyzed. Patient characteristics are summarized in Table 1. The median patient age was 58 years (range, 5–69) and 4 patients were younger than 18 years. Most patients had grade III–IV aGVHD, with gut and/or liver involvement. All patients received MSCs as the first rescue therapy after corticosteroids, with the exception for one patient who received prior treatment with mycophenolate mofetil (that was still ongoing by the time of MSC therapy). Twenty patients received MSCs for steroid-refractory and 13 for steroid-dependent aGVHD. Median time from grade II–IV aGVHD diagnosis to first MSC infusion was 16 days (range 3–76 days).

**Table 1:** Patient characteristics (*n* = 33)

Patients		
Patient age at inclusion, median (range), years	58	(5–69)
<18 years, *n* (%)	4	(12)
18–50 years, *n* (%)	6	(18)
> 50 years, *n* (%)	23	(70)
Patient gender, male, *n* (%)	24	(73)
Primary disease, *n* (%)		
Acute myelogenous leukemia	14	(42)
Myelodysplastic syndrome	7	(21)
Other hematological malignancy^*^	12	(36)
**Transplantation**		
Conditioning regimen, *n* (%)		
Myeloablative	8	(24)
Reduced intensity	25	(76)
Stem cell source, *n* (%)		
PBSC	30	(91)
UCB	3	(9)
Type of donor (HSC), *n* (%)		
Related	10	(30)
Unrelated	23	(70)
HLA-matched ^¶^	23	(70)
HLA-mismatched ^¶^	9	(27)
Female donor for male recipient	5	(15)
GVHD prophylaxis		
CyA/tacro + MTX	6	(18)
CyA/tacro + MMF	16	(48)
CyA	8	(24)
Others^§^	3	(9)
Pre-transplant ATG	17	(52)
**AGVHD**		
Time from alloHCT to grade II–IV aGVHD diagnosis, median (range), days	80	(8–358)
Late (>100 days) aGVHD after alloHCT, *n* (%)	9	(27)
aGVHD after DLI, *n* (%)	4	(12)
Overall grade aGVHD at inclusion, *n* (%)		
Grade II	9	(27)
Grade III	15	(45)
Grade IV	9	(27)
Organ (s) involved in aGVHD, *n* (%)		
Skin	17	(52)
GI tract	27	(82)
Liver	12	(36)
Multiple organs (≥2)	18	(55)
Single organ: Skin only	5	(15)
Gut only	10	(30)
Liver only	0	(0)
Indication for MSC therapy, *n* (%)		
Steroid-refractory aGVHD	20	(61)
Steroid-dependent aGVHD	13	(39)
MSCs		
MSC as 1st line rescue therapy for cortico-resistant/dependent aGVHD, *n* (%)	32	(97)
Time from grade II–IV aGVHD diagnosis to first MSC infusion, median (range), days	16	(3–76)
First MSC infusion, dose, *n* (%)		
1–2 × 10^6^ cells/kg recipient’s bodyweight	20	(61)
3–4 × 10^6^ cells/kg recipient’s bodyweight	13	(39)
2nd MSC infusion, *n* (%)^¥^	8	24

AGVHD, acute graft-versus-host disease; alloHCT, allogeneic hematopoietic cell transplantation; ATG, anti-T cell globulin; CyA, cyclosporine A; DLI, donor lymphocyte infusion; GI, gastro-intestinal; GVHD, graft-versus-host disease; HSC , hematopoietic stem cells; MSC, multipotent mesenchymal stromal cells; MMF, mycophenolate mofetil; MTX, methotrexate; PBSC, peripheral blood stem cells; UCB, umbilical cord blood.

^*^Other malignancies were acute lymphoblastic leukemia (*n* = 3), chronic lymphocytic leukemia (*n* = 3), non-Hodgkin lymphoma (*n* = 2) and multiple myeloma (*n* = 4).

^¶^Donor/ recipient HLA-matching status missing for one patient.

^¥^5 patients received a second dose of 1–2 × 10^6^ MSCs/kg and 3 received a second dose of 3–4 × 10^6^ MSCs/kg.

^§^ Other GVHD prophylaxes included combination of tacrolimus + sirolimus (2 patients) and *ex-vivo* T-cell depletion (1 patient).

**Figure 1:**
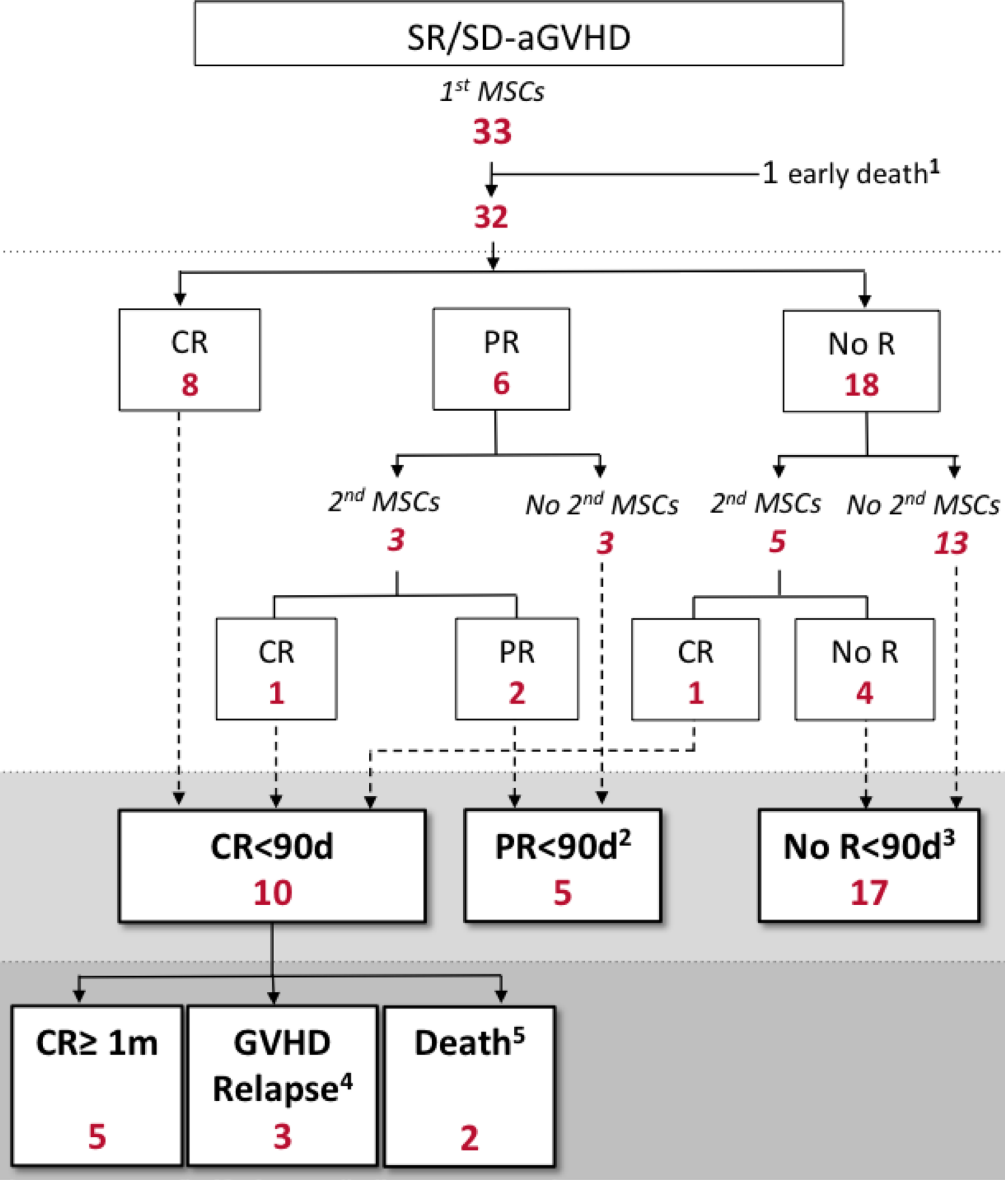
MSC administration and aGVHD response to MSC therapy SR/SD-aGVHD, steroid refractory/dependent acute graft-versus-host disease; CR, complete response of aGVHD; CR≥ 1m, complete response lasting more than 1 month; MSC, mesenchymal stromal cell; No R, no response of aGVHD; PR, partial response of aGVHD; <90d, within 90 days after first MSC infusion. ^1^One patient died of diffuse alveolar damage within 24 hours after MSC infusion. ^2^Among the 5 patients who achieved PR<90d with MSC therapy, 1 maintained PR for at least 1 month whereas 3 experienced aGVHD worsening and 1 died of TTP within the month after achieving PR. ^3^ Among the 17 patients with no response to MSC therapy: 2 died less than 10 days after first MSC infusion (1 of infection and 1 of aGVHD); 6 did not receive rescue therapy other than a second MSC infusion; and 9 received a median of 1 (range 1–3) additional line (s) of immunosuppressive therapy (including anti-T-cell globulins, mycophenolate mofetil, mTOR inhibitors, anti-TNFα agents) with the first of them initiated after a delay of less than 10 days in 3 patients. Among these 9 patients, 4 were successfully rescued with subsequent salvage therapies. For the two patients who died less than 10 days after first MSC infusion, because the cause of death was directly or indirectly (infection) related to aGVHD, they were considered as non-responders to MSC therapy. ^4^Three patients experienced aGVHD recurrence 16, 23 and 25 days after achieving CR. Two had grade II and one had grade III aGVHD. ^5^Two patients died of infections within the month after achieving CR.

Twenty patients received a first dose of 1–2 × 10^6^ MSCs/kg and 13 patients received a first dose of 3–4 × 10^6^ MSCs/kg, depending on the period when they were included in the study (protocol amendment in January 2010 after an interim analysis indicating poor results with 1–2 million MSCs/kg, see Methods). There was no difference between these two consecutive subgroups of patients in terms of baseline patient and aGVHD characteristics, with the exception of use of pre-transplant ATG (with a lower proportion of patients having received pre-transplant ATG in the 3–4 × 10^6^ MSCs/kg cohort) (see Supplementary Table 1). In case of failure to achieve a complete response after first MSC infusion, a second infusion of MSCs could be administered, depending on the attending physician’s judgement (see Methods). Eight patients received a second MSC infusion (Figure 1). MSCs for the second infusion were obtained from either the same (*n* = 4) or a different (*n* = 4) donor as for the first infusion and were administered at a dose equivalent to the first one. Hence, among the 20 patients who received a first 1–2 × 10^6^/kg dose of MSCs, 5 received a second equivalent dose; and among the 13 patients who received a first 3–4 × 10^6^/kg dose of MSCs, 3 received such a second dose.

### MSC characteristics

Altogether, a total of 41 MSC infusions from 15 donors were administered. Bone marrow samples were collected and MSCs were expanded as previously described [29]. Median age of MSC donors was 25 years (range, 18–52) and 83% of MSC products were collected from male donors. The median population doubling level of MSCs between passage 1 and passage 3 (harvest) was 4.9 (range 4.1–6.6) and the median MSC viability after thawing was 80% (range 54–96%).

### AGVHD response to MSC therapy

Among the 33 evaluable patients, one patient died within 24 hours after first MSC infusion (see below, safety issue) and was censored for the efficacy analysis.

**Figure 2:**
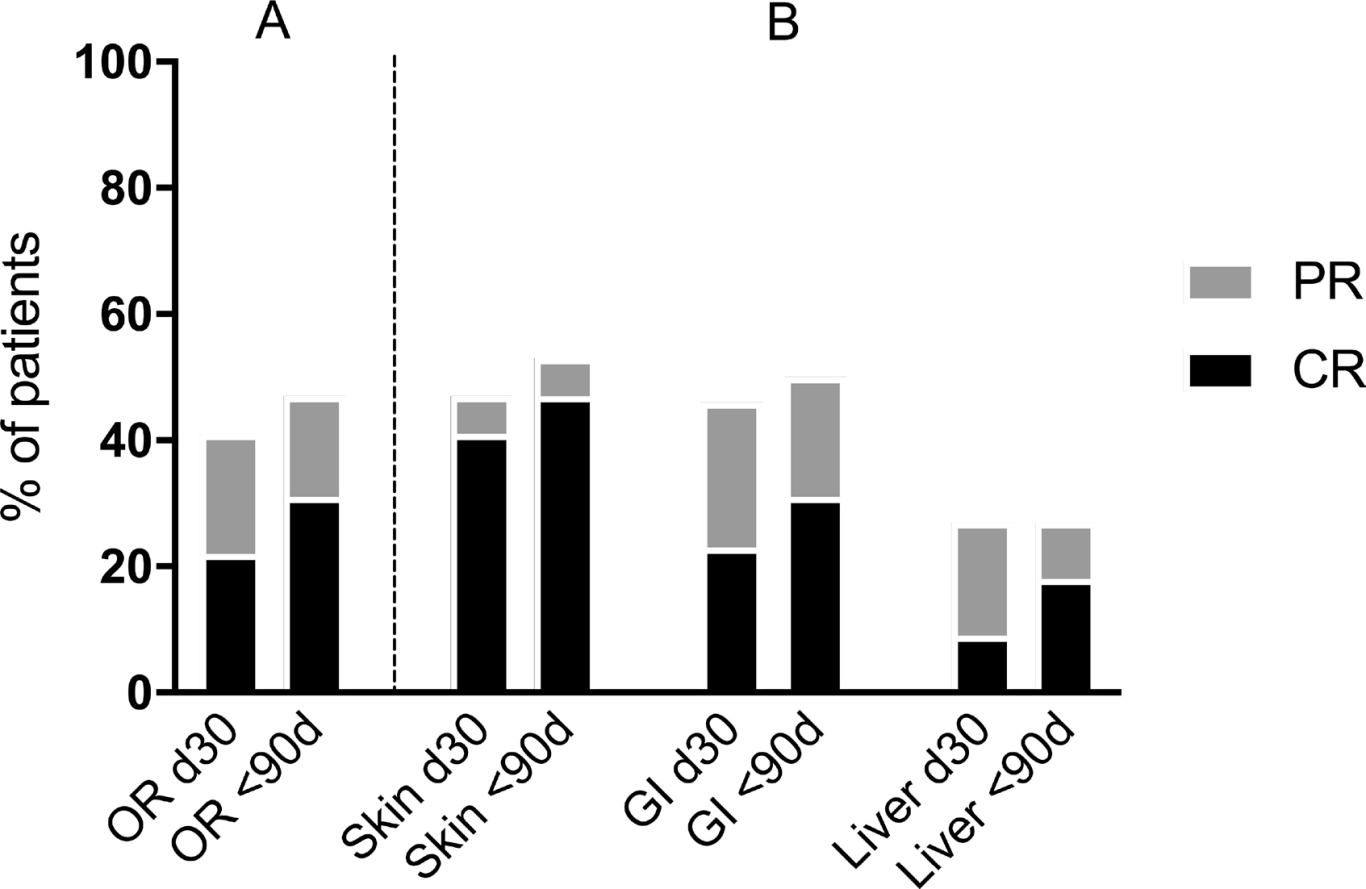
Response of aGVHD at day 30 (d30) and within the 90 days (<90d) after initiation of MSC therapy (**A**) overall response, (**B**) organ specific response (skin *n* = 17; GI tract *n* = 26; liver *n* = 11). CR, complete response of aGVHD; GI, gastrointestinal tract; PR, partial response of aGVHD. The differences between skin, GI and liver response rates (CR, PR and overall response) were not statistically significant, both at day 30 (d30) and within the 90 day-period (<90d) after first MSC infusion (*p* = NS).

Thirteen patients (40.6%, 95% CI: 25.5–57.7%) achieved an overall response at day 30 after first MSC infusion (ORd30), while 15 patients (46.9%, 95% CI: 30.9–63.5%) reached an overall response within 90 days after initiation of MSC therapy (OR<90d) (Figure 2). The corresponding complete response rates were 21.9% (95% CI: 11–38.7%, 7 patients) at day 30 (CRd30) and 31.2% (95% CI: 17.9–48.6%, 10 patients) within the 90 days after initiation of MSC therapy (CR<90d). Responses by organ (regardless of the number of organs involved in a particular patient) at day 30 and within 90 days after initiation of MSC therapy is depicted in Figure 2. Median time from first MSC infusion to reach at least a partial response (PR<90d) was 7 days (range, 3 to 10 days) and to reach CR<90d was 22 days (range, 3 to 61 days).

**Figure 3:**
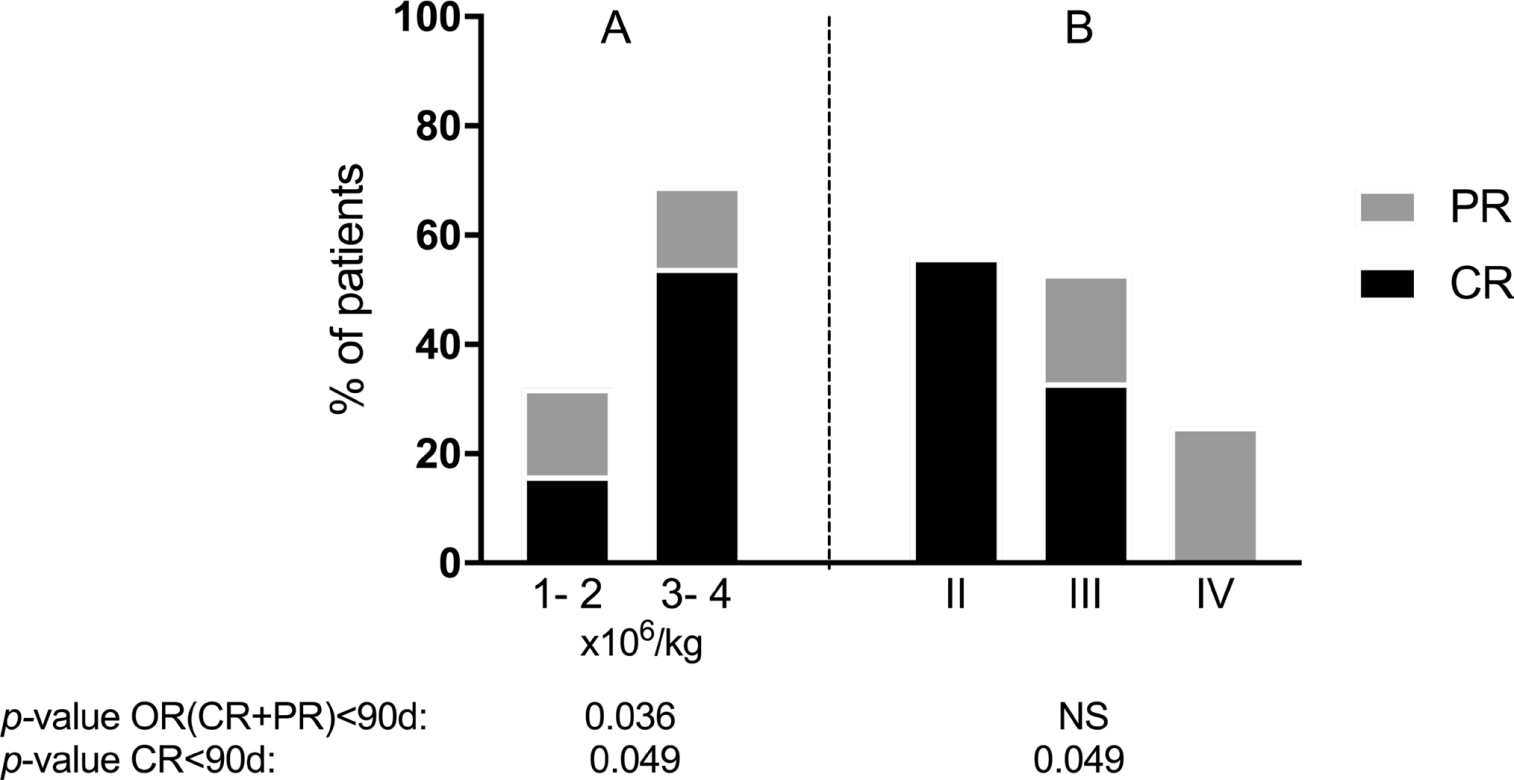
Response of aGVHD within the 90 days after initiation of MSC therapy according to (**A**) MSC dose for first infusion and (**B**) aGVHD grade.

We further analysed potential associations between patient characteristics at study entry and aGVHD response to MSC therapy, assessed as both OR<90d and CR<90d. Results of the univariate analysis are illustrated in Supplementary Table 2. Compared to patients who received 1–2 × 10^6^ MSCs/kg, patients who received 3–4 × 10^6^ MSCs/kg had a better chance of achieving both OR<90d (69.2% *versus* 31.6%, *p* = 0.036) and CR<90d (53.8% *versus* 15.8.%, *p* = 0.049) (Figure 3A). Grade of aGVHD was also associated with CR<90d but not with OR<d90 (Figure 3B). No other factor was associated with response to MSC therapy. Regarding MSC donors, we noted variable types of response among recipients of MSCs originating from a same donor.

Among the 10 patients who achieved CR<90d with MSC therapy, 5 maintained complete remission for at least 1 month (CR ≥ 1 m), while 3 experienced aGVHD recurrence and 2 died (both due to infections) within the month after having obtained CR<90d (Figure 1). Of note, all 5 patients who maintained CR ≥ 1m had received a dose of 3–4 × 10^6^ MSCs/kg.

### Survival

**Figure 4:**
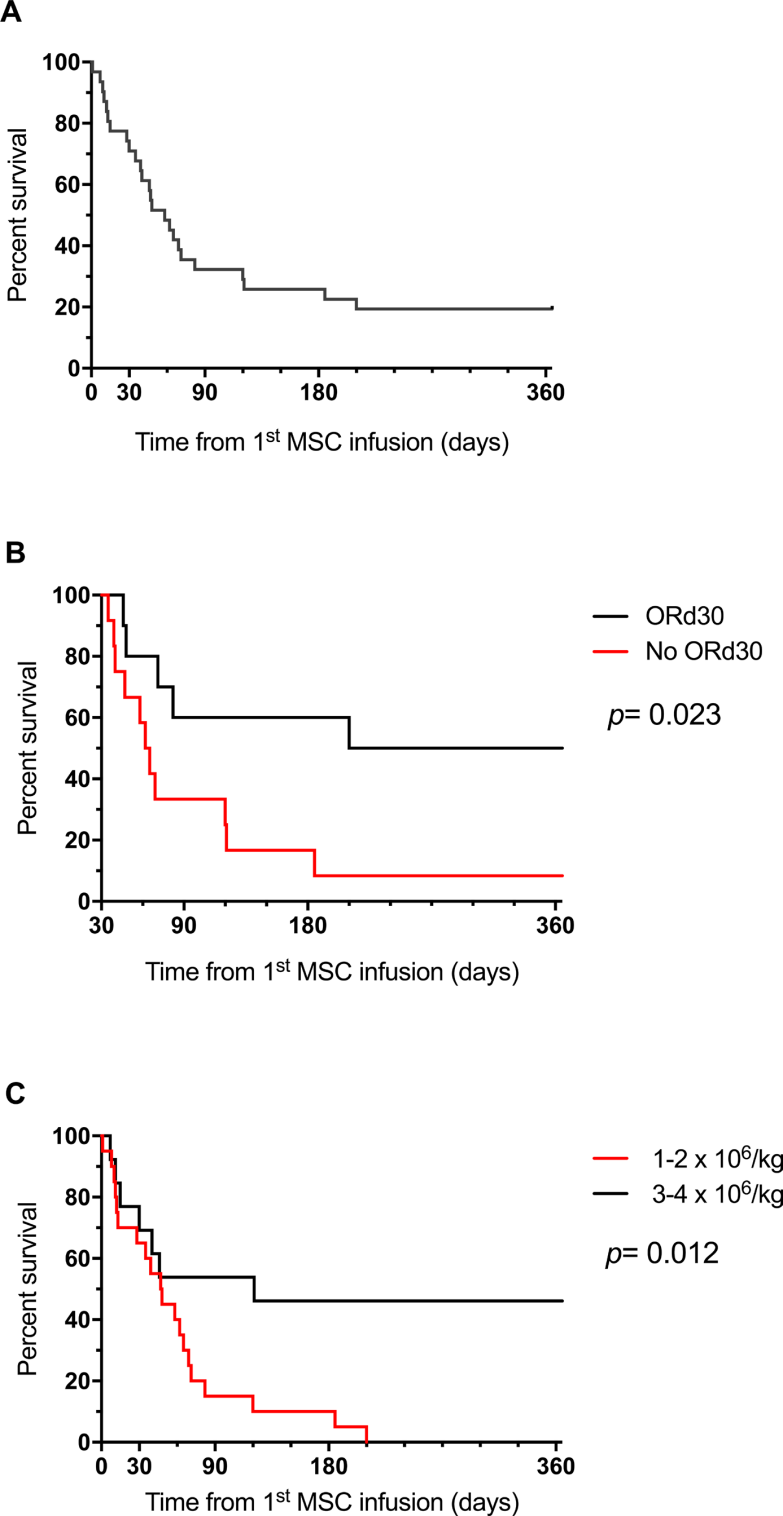
Survival curves: (**A**) OS for the global cohort (**B**) landmark analysis at d30 according to ORd30 and (**C**) OS according to MSC dose for first infusion.

The 90-day and 1-year overall survival (OS) rates after initiation of MSC therapy were 30.3% (95% CI: 18.1–50.8%) and 18.2% (95% CI: 8.82–37.5%), respectively (Figure 4A). Infections and persistence of aGVHD were the leading causes of deaths (Table 2).

**Table 2:** Causes of death according to delay from first MSC infusion

	Mortality d0-d90	Mortality d91–d365
aGVHD	7	0
Infection	11	2
Relapse	2	2
Other	3^*^	0

^*^One patient died of diffuse alveolar damage, one of worsening of previous cardiac failure and one of thrombotic thrombocytopenic purpura.

Landmark survival analysis at d30 revealed that patients who achieved ORd30 had better 1-year OS than non-responders (50% *versus* 7.7%, *p* = 0.023) (Figure 4B). Landmark analysis at day 90 was not performed because of the low number of patients still alive at that time. Of note, the 5 patients who achieved CR≥ 1m within the 90 days after MSC therapy were alive at 1 year.

Interestingly, patients who received a MSC dose of 3-4 × 10^6^/kg experienced significantly better 1-year OS than patients who received 1–2 × 10^6^ MSCs/kg (46.2% *versus* 0.0%, *p* = 0.012) (Figure 4C).

### Safety data and other clinical outcomes

A total of 41 MSC infusions were administered. Three patients experienced fatal serious adverse events. One patient with grade IV aGVHD died with acute respiratory distress syndrome within 24 hours after receiving a dose of 1.7 × 10^6^ MSCs/kg. Autopsy revealed diffuse pulmonary alveolar damages without evidence of infection or infiltration by leukemic cells. Another patient with grade II aGVHD developed fatal thrombotic thrombocytopenic purpura (TTP) at day 6 after MSC infusion at a dose of 1.4 × 10^6^/kg (no autopsy performed). The patient was concomitantly treated with ciclosporine and voriconazole. In both cases, the relationship with MSC infusion could not be formally excluded. Another patient with severe underlying cardiopathy died of progressive heart failure at day 71 after MSC infusion (at a dose of 2 × 10^6^ MSCs/kg). This was considered as not related with MSC therapy by the investigators.

During the 1-year follow-up period after initiation of MSC therapy, a total of 81 serious infectious events were recorded (51 bacterial, 16 viral, 12 fungal and 2 parasitical events). The 1-year cumulative incidence of a first serious infectious event after initiation of MSC therapy was 71.9% (95% CI: 52.9–84.3%) and most of them occured during the first 90 days after MSC therapy (Supplementary Figure 1). The cumulative incidence of disease relapse at 1 year after MSC infusion was 18.8% (95% CI: 7.6–33.7%) (Supplementary Figure 1). Median time from MSC infusion to relapse was 150 days (range 14–299 days). No secondary malignancy was observed during the first year after MSC therapy, with the exception of one case of EBV-driven posttransplant lymphoproliferative disease at day +59 after MSC therapy.

## DISCUSSION

Steroid-refractory aGVHD remains one of the great challenges after alloHCT. In this multicenter prospective study, we report the results of a series of 33 evaluable patients with SR/SD-aGVHD treated with 1 or 2 intravenous infusion(s) of BM-derived MSCs from third party donors The study was conducted within the setting of a single academic clinical-grade cell production facility, thereby ensuring a homogeneous MSC manufacturing process. MSCs were expanded in FBS-supplemented medium, cultured only up to 3 passages and cryopreserved [29]. All patients received MSCs as the first rescue therapy after corticosteroids, with the exception for one patient who received prior treatment with mycophenolate mofetil (that was still ongoing by the time of MSC therapy). In these conditions, the administration of 1–2 dose(s) (each dose ranging from 1 to 4 x 10^6^ cells/recipient’s kg body weight) of MSCs resulted in an overall response rate of 40.6% at day 30 and 46% within 90 days. The corresponding complete response rates were 21.6% at day 30 and 30% within 90 days.

These response rates appeared to be less optimistic than those reported in most previous studies having tested MSC therapy for SR/SD-aGVHD [16, 18–21, 23, 25]. The pioneer study by K. LeBlanc *et al.*, one of the largest studies thus far, reported overall and complete response rates of 70.9% and 54.5%, respectively [21]. High response rates were similarly observed in a number of other recent studies [16, 18–20, 23, 25]. Although a direct comparison of our results with these previous studies is not possible because of variable definitions and timing of response evaluation, we can only speculate about factors that may have negatively influenced the response rates in our cohort. First, the majority of our patients were older than 50 years (median age 58 years), while most « positive » studies have enrolled children and/or younger adults [16, 18, 19, 21, 23, 25]. It has previously been suggested that patient age could impact the efficacy of MSC therapy, with younger age tending to be associated with better clinical response [21, 30]. Moreover, patients in our study composed a challenging population with a majority of them suffering from severe (grade III or IV) aGVHD and with visceral organ involvement at baseline. Both of these factors have been associated with poor response to MSC therapy [30, 31]. Our cohort also consisted of a significant proportion of patients with corticosteroid-dependent aGVHD and the median time from grade II-IV aGVHD diagnosis to first MSC infusion was longer than reported in most previous studies. Nevertheless, none of these parameters was associated with response to MSC therapy in our analysis. The infusional schedule in our protocol consisted in only 1–2 MSC administration(s), inspired by the pioneer study by K. LeBlanc *et al.* in which about 90% of patients were treated with solely 1–2 infusions [21]. In contrast, multiple MSC doses were generally infused in most recent positive studies [16, 18–20, 25]. Recent findings have indicated that a higher number of infusions might be required to reverse the course of SR/SD-aGVHD [16, 25, 30, 32, 33]. Hence, we cannot exclude that continued therapy beyond the initial 1–2 MSC infusions might have further improved the response rate in our cohort, in particular the CR rate.

In the setting of 1-2 MSC administration(s), we also observed a higher proportion of responders and of complete responders among patients who received the highest dose (3–4 × 10^6^ instead of 1–2 × 10^6^ cells/kg). The observed difference in response rates can probably not be attributed to other aGVHD baseline characteristics, since those confounding factors were well balanced between the two dose groups. To the best of our knowledge, no other study has ever reported a dose–response relationship, not even after using mega-doses of MSCs [34]. However, in most previous studies, the majority of patients received multiple MSC infusions [16, 18–20, 25] and it is not excluded that the multiplicity of MSC administrations might have balanced the dose-effect of the first infusion. Nevertheless, our results have to be interpreted with caution, regarding the small number of patients.

In our study, only half of the complete responders to MSC therapy maintained their CR beyond 1 month. This is in line with the results observed by LeBlanc *et al.* reporting that, among 30 complete responders at 6 weeks after MSC infusion, only 19 maintained prolonged response [21]. Higher rates of sustained response were recently reported by Sanchez *et al.*, using multiple MSC infusions [20]. The latter study, analyzing the outcomes of 24 patients transfused with 4 sequential MSC infusions, observed that only 2 of the 17 responders to MSCs experienced a recurrence at day 60 after treatment initiation. Clearly, further studies are needed to assess whether continued therapy beyond the initial 1–2 doses might be beneficial for maintenance of response. However, preliminary results of the sole yet completed randomized phase III placebo-controlled trial having used multiple MSC administrations (2 × 10^6^ MSCs/kg, twice weekly for 4 consecutive weeks) failed to report more durable responses with MSC therapy compared to placebo, in addition to institutionally selected second line treatment [26].

We observed a low OS at 1 year after initiation of MSC therapy (18.2%; 95% CI: 8.82–37.5%), emphasizing the fact that SR/SD-aGVHD is associated with a dismal outcome. Similarly, VonDalowski *et al.* recently reported a 1-year OS of 19% (95% CI, 9%–29%) in a retrospective analysis of 58 adult patients treated with MSC therapy for SR/SD-aGVHD [33]. Our results were also in line with survival reported by K. Leblanc *et al.* in the subgroup of adult patients (26% 2-year OS [95% CI, 10%–42%]) [21]. Nevertheless, the survival rate we observed in our cohort contrasted with the 1-year survival of about 40–50% reported in some concomitant studies assessing MSC or other non-MSC strategies (such as anti-T cell globulins) for controlling SR/SD-aGVHD [18, 22, 35]. One hypothesis could be that our patients composed a very challenging population. For example, patients with aGVHD after alloHCT with HLA-mismatched donors, after donor lymphocyte infusion (DLI) and with late acute GVHD were included in our study but excluded in some others [35]. We also had high proportions of patients > 50–60 years or suffering from severe visceral (gut or liver) aGVHD, and both of these factors have been described to be associated with poor outcomes [31, 35]. Interestingly, in our study, very poor outcome was especially observed in patients who received 1–2 × 10^6^ MSCs/kg, with none of them surviving at 1 year. In contrast, patients receiving 3–4 × 10^6^ MSCs/kg experienced a significantly better survival of 46% at 1 year (thus in the range of the other studies [18, 22, 35]. This could be interpreted in line with the positive association between MSC dose and response rate of aGVHD, as described above. Accordingly, we observed that achieving response at day 30 after MSC therapy resulted in improved OS. This was in agreement with previous reports suggesting that clinical response of aGVHD to therapy at day 28 correlates well with non-relapse mortality and OS [36–38].

The leading causes of death in our study were persistence of aGVHD and infections. Regarding infectious events, more than two thirds of our patients experienced at least one serious infection within the first year after initiation of MSC therapy. The relationship between MSC infusions and infections could not be established in this study, since there was no comparative control group of SR/SD-aGVHD patients not treated with MSC. Infectious events are frequent complications in patients with SR/SD-aGVHD [39, 40]. Interestingly, comparable cumulative incidences of infections were recently reported by García-Cadenas, in a retrospective study of 127 adult patients with SR/SD-aGVHD treated with inolimomab or etanercept [40]. In a prospective study, Zhao *et al.* compared infection rates between MSC recipients and non-MSC control patients and did not find significant differences [19].

Regarding possible acute toxicity of MSC therapy, we observed 2 early fatal events. The first patient died because of diffuse pulmonary alveolar damages within 24 hours after MSC infusion and the second patient died because of TTP at day 6 after MSC infusion. Although we cannot formally exclude a relationship with the MSC infusions, the causability remains unknown since these complications are frequently observed in transplanted patients with SR/SD-aGVHD who are in poor general condition and who classically receive a lot of medications. Based on our published experience with MSC therapy for other indications than SR/SD-aGVHD, we have never observed similar early complications in more than 200 treated patients (with MSCs produced and administered in the same way as here) [29, 41]. In the setting of SR/SD-aGVHD, numerous previous reports have indicated an excellent safety of intravenous MSC infusions [16–21, 25, 26]. A recent meta-analysis of prospective studies summarized toxicity outcomes related to MSC treatment for a range of conditions (including inflammatory diseases, stroke, cardiomyopathy, healthy volunteers and aGVHD) [42]. The meta-analysis of randomized controlled trials (8 studies including 321 patients) did not detect any association between MSC administration and acute infusional toxicity, organ toxicity or death [42]. To our knowledge, no case of TTP after MSC administration has been previously reported.

Recent insights into MSC biology have led to the development of novel strategies aimed at improving MSC-based therapy. These include, among others, donor selection basing on biological parameters [43, 44], use of pooled donor batches rather than single-donor derived units [23] and infusion of MSC-derived exosome suspensions rather than complete cellular products [45, 46]. Whether these approaches will improve MSC efficacy against SR/SD-aGVHD will have to be demonstrated in the future.

In conclusion, controversies currently remain regarding the real clinical effectiveness of MSC therapy in SR/SD-aGVHD. In this study, response rates and OS were less optimistic than those reported in some previous studies. Since our study was a single-arm prospective study, it is not possible to say whether the poor outcome we observed in our cohort was due to poor efficacy of MSC therapy or to the fact that our cohort constituted a particularly challenging population. Phase III studies comparing MSC with non-MSC treatment are urgently needed. Moreover, standardization in MSC production and protocols for administration are fundamental prerequisites to optimize their use in randomized controlled studies. Whether increasing doses for initial infusions or performing multiple sequential infusions might improve the rate and the durability of response to MSC therapy has to be explored in further studies.

## MATERIALS AND METHODS

### Patient eligibility and study design

Patients developing aGVHD after alloHCT were eligible, regardless of age, conditioning regimen, graft source and type of donor. Patients developing aGVHD after donor lymphocyte infusion were also considered for this study. Biopsy for confirmation of aGVHD was recommended, but not required. AGVHD was graded by physicians at individual centers according to the Glucksberg modified criteria [47]. AGVHD refractoriness to corticosteroids was defined as progression after 3 days, no improvement after 7 days, or absence of complete resolution after 14 days of treatment with 2 mg/kg/day methylprednisolone or equivalent. AGVHD dependence on corticosteroids was defined as aGVHD recurrence during steroid taper. Patients could have received any other line of immunosuppressive therapy in addition to steroids for treating aGVHD, but no new treatment started within the month preceding MSC infusion. These treatments could be continued or discontinued at the time of initiation of MSC therapy, based on investigator’s judgment. Patients with relapsing or progressing malignancy, HIV infection or active uncontrolled infection were not eligible for this study. Patients or their legal guardians provided written informed consent to enroll in the study.

The study started recruitment in January 2008. All patients received at least one MSC infusion at a dose of 1–2 or 3–4 million MSCs/kg body weight, depending on the period when they were included in the study: patients included before January 2010 received a dose of 1–2 million MSCs/kg, while those included after January 2010 received a dose of 3-4 million MSCs/kg (protocol amended on January 7th 2010, after an interim analysis indicating poor results with 1-2 million MSCs/kg). In case of failure to achieve a complete response within at least 10 days after first MSC infusion, a second infusion of MSCs at an equivalent dose could be administered, depending on the attending physician’s judgement. Patients were also allowed to receive any other novel line of immunosuppressive agent in such cases. However, they would be considered off study for aGVHD response analysis by that time (and registered as « non responders »). Addition of novel treatment less than 10 days after MSC infusion was discouraged.

The protocol was approved by the respective ethics review boards of all participating centers and the study was conducted in accordance with the Declaration of Helsinki. This clinical trial was registered at www.clinicaltrials.gov (#NCT00603330).

### MSC production, thawing and administration

BM-derived MSCs were collected from third-party healthy volunteer donors at the CHU of Liège (Liège, Belgium). Written informed consent was obtained from each donor and the MSC harvest protocol was approved by the institutional ethics review board. MSCs were expanded and stored in the clinical-grade cell production facility of the Laboratory of Cell and Gene Therapy (LCGT) at the University of Liège (Liège, Belgium), as previously described [29]. Briefly, MSCs were cultured in Dulbecco’s Modified Eagles Medium–Low Glucose with Glutamax supplemented with 10% gamma-irradiated FBS in a normoxic and humidified atmosphere. MSC were harvested after three passages (14 days of primary culture and 2 passages of 7 days each), and frozen in a 10% dimethyl sulfoxide (DMSO)-containing solution in sterile freezing bags. The population doubling level was calculated from the time the first adherent mononuclear cell population was harvested (3.32 (log Y−log I), where Y = number of cells harvested and I = number of cells inoculated at passage 1).

The European Society for Blood and Marrow Transplantation (EBMT) release criteria were prospectively applied to deliver cryopreserved MSCs for clinical use, although the cells were also compliant with the ISCT criteria [29]. Cryopreserved MSC aliquots were chosen based on cell counts determined at harvest (before freezing), to offer a MSC dose adapted to each patient’s body weight (see above). No other parameter was considered for batch selection.

Cryopreserved MSCs were thawed and diluted at the LCGT for patients treated at the University of Liège Hospital (CHU of Liège, Liège, Belgium) or were transferred in a temperature-monitored liquid nitrogen container and thawed at local labs for patients treated in other centers. MSCs were thawed according to a uniform protocol provided by the LCGT. Thawed cell numeration and viability were controlled by centers and MSCs were delivered for clinical use only if cell recovery was adequate for dose specifications and if their viability was >50%.

MSC administration to the patient had to be performed within 1 hour of thawing. Cells were given intravenously, through a central venous catheter. Patients were systematically premedicated with 2mg/kg methylprednisolone and an anti-histaminic drug.

### AGVHD response to MSC therapy

AGVHD grade was prospectively recorded on days 0, +3, +7, +10, +30, +60 and +90 after first MSC infusion using the Glucksberg modified criteria [47]. Definitions used for aGVHD response to MSC therapy are summarized in Table 3. We chose to report aGVHD response using two definitions: (1) overall response at day 30; and (2) best response within the 90-day period after first MSC infusion [37, 38]. Response were reported both on the basis of organ-specific and overall grade response. If death occured before 30 days, response to MSC treatment at day 30 was recorded as the response on the date of death. Patients were considered to achieve an overall response (OR) if they obtained either a complete response (CR) or a partial response (PR). Finally, the duration of response was also assessed in patients who achieved a CR within 90 days (CR < 90d) and patients were reported to have a sustained CR (CR ≥ 1m) if they remained alive in CR for at least 1 month.

**Table 3:** Response of aGVHD to MSC therapy: definitions

Types of response	Overall grade response^*^
**Complete response (CR)**	Resolution of all signs of aGVHD (aGVHD overall grade = 0)^*^
**Partial response (PR)**	Decrease of the aGVHD overall grade by at least 1 grade as compared with baseline overall grade^*^
**Overall response (OR)**	Achievement of either CR or PR
**No response (No R)**	Not fulfilling criteria for CR or PR
**Response at day 30 after first MSC infusion**	
**CRd30**	Achievement of CR at day 30 after first MSC infusion
**PRd30**	Achievement of PR at day 30 after first MSC infusion
**ORd30**	Achievement of either CRd30 or PRd30
**No Rd30**	Not fulfilling criteria for CRd30 or PRd30
**Best response observed within the 90-day period after first MSC infusion**	
**CR<90d**	Achievement of CR as best response at least at one time-point within 90 days after first MSC infusion
**PR<90d**	Achievement of PR as best response at least at one time-point within 90 days after first MSC infusion
**OR<90d**	Achievement of either CR<90d or PR<90d
**No R<90d**	Not fulfilling criteria for CR<90d or PR<90d
**1-month maintenance of CR within the 90-day period after first MSC infusion**	
**Sustained complete response (CR ≥ 1m)**	CR maintained for at least 1 consecutive month

^*^Organ (skin, gut, liver) specific responses were also assessed according to the initially affected organs at baseline. CR was defined as resolution of all signs of aGVHD in the specific organ (aGVHD stage in the specific organ = 0). PR was defined as decrease of the aGVHD stage in the specific organ by at least 1 stage as compared with baseline stage.

Sub-analyses of aGVHD response according to patient characteristics at study entry were performed using OR<90d and CR<90d. We chose to use OR<90d and CR<90d rather than ORd30 and CRd30 for these analyses, because of previous reports having shown delayed response of aGVHD to MSC therapy, particularly for CR (occuring even after d30 in some cases) [22].

### Other clinical outcomes

Other clinical outcomes included overall survival and disease relapse at 1 year after initiation of MSC therapy. Serious infectious events were also registered, as previously defined [48]. Safety was also carefully monitored.

### Statistical analysis

Comparisons were performed by using Chi-square, Fisher’s exact or Mann-Whitney rank sum tests. Response rates were expressed as proportions. Overall survival was estimated with the Kaplan-Meier method and cumulative incidence functions were estimated for competing risk analyses. Death was considered as a competing risk for relapse. Death and relapse were considered as competing risks for infection. Comparisons of survival in groups were performed by log-rank test. Statistical analyses were performed using GraphPad Prism (GraphPad Software, San Diego, CA) and SAS version 9.3 (SAS Institute, Cary, NC, USA). Statistical significance was set at a level of *p* < 0.05.

## SUPPLEMENTARY MATERIALS FIGURE AND TABLES



## Abbreviations

aGVHD: acute graft-versus-host disease
alloHCT: allogeneic cell transplantation
BM: bone marrow
CR: complete response of aGVHD to MSC therapy
FBS: fetal bovine serum
HLA: human leukocyte antigen
ISCT: International Society for Cellular Therapy
LCGT: Laboratory of Cell and Gene Therapy
MSC: multipotent mesenchymal stromal cell
OR: overall response of aGVHD to MSC therapy
OS: overall survival
PR: partial response of aGVHD to MSC therapy
SR/SD-aGVHD: steroid-refractory or steroid-dependent aGVHD (SR/SD-aGVHD)
TTP: thrombotic thrombocytopenic purpura
