# Supplementary material for: Infusion of bone marrow derived multipotent mesenchymal stromal cells for the treatment of steroid-refractory acute graft-versus-host disease: a multicenter prospective study
Source: Oncotarget. 2018 Apr 17;9(29):20590–604. doi: 10.18632/oncotarget.25020 (PMC5945536; doi:10.18632/oncotarget.25020)
Supplement: Supplementary file 1 [file oncotarget-09-20590-s001.pdf]

Infusion of bone marrow derived multipotent mesenchymal stromal cells for the treatment of steroid-refractory acute graft-versus-host disease: a multicenter prospective study

SUPPLEMENTARY MATERIALS

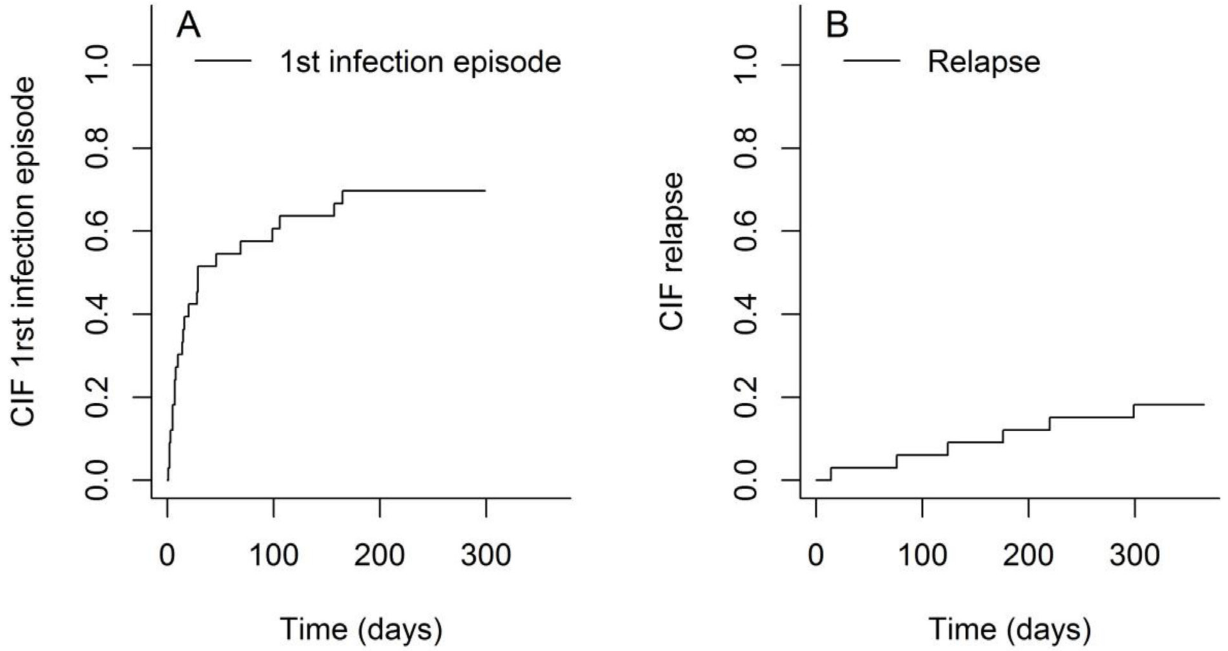

Supplementary Figure 1: Cumulative incidences of first infectious event (**A**) and relapse (**B**) after initiation of MSC therapy.

Supplementary Table 1: Comparison of the two 2 consecutive cohorts of patients receiving 1–2 or 3–4 × 10^6 MSCs/kg per dose

	**1–2 × 10^6 MSC/kg *n* = 20**	**3–4 × 10^6 MSC/kg *n* = 13**	***p* value**
Patient age, median (range), years	57 (5–69)	60 (6–67)	0.74
Patient gender, male, *n* (%)	15 (75)	9 (69)	0.99
Myeloablative conditioning regimen, *n* (%)	4 (20)	4 (31)	0.68
Stem cell source, PBSC, *n* (%)	18 (90)	12 (92)	0.99
Unrelated donor, *n* (%)	15 (75)	8 (61)	0.46
HLA-mismatched donor*, *n* (%)	6 (30)	3 (23)	0.99
Female donor for male recipient, *n* (%)	2 (10)	3 (23)	0.36
GVHD prophylaxis			0.29
CyA/tacro + MTX	2 (10)	4 (31)	
CyA/tacro + MMF	10 (50)	6 (46)	
Others	8 (40)	3 (23)	
Pre-transplant ATG, *n* (%)	14 (70)	3 (23)	0.0084
Time from alloHCT to grade II–IV aGVHD diagnosis, median (range), days	82 (8–358)	80 (11–258)	0.90
Overall grade aGVHD, *n* (%)			0.65
II	4 (20)	5 (38)	
III	10 (50)	5 (38)	
IV	6 (30)	3 (23)	
Multiple organ involvement, *n* (%)	12 (60)	6 (46)	0.44
Indication for MSC therapy, *n* (%)			0.41
Steroid-refractory aGVHD	11(55)	9 (69)	
Steroid-dependent aGVHD	9 (45)	4 (31)	
Time from grade II-IV aGVHD diagnosis to 1st MSC infusion, median (range), days	25 (3–76)	11 (3–39)	0.18

AGVHD, acute graft-versus-host disease; alloHCT, allogeneic hematopoietic cell transplantation; ATG, anti-T cell globulin; CyA, cyclosporin A; DLI, donor lymphocyte infusion; GI, gastro-intestinal; GVHD, graft-versus-host disease; HSC; hematopoietic stem cells; MSC, multipotent mesenchymal stromal cells; MMF, mycophenolate mofetil; MTX, methotrexate; PBSC, peripheral blood stem cells.

*Donor/recipient HLA-matching status missing for one patient who received 1–2 × 10^6 MSC/kg.

Supplementary Table 2: Univariate analysis of OR<90d and CR<90d according to patient and aGVHD characteristics at baseline

	OR<90d		CR<90d	
	***n* (%)**	***p* value**	***n* (%)**	***p* value**
Patient age (years)		0.74		0.99
<18	1 of 4 (25)		1 of 4 (25)	
18–50	3 of 5 (60)		1 of 5 (20)	
>50	11 of 23 (47.8)		8 of 23 (34.8)	
Patient gender		0.12		0.68
Female	2 of 9 (22.2)		2 of 9 (22.2)	
Male	13 of 23 (56.5)		8 of 23 (34.8)	
Conditioning regimen		0.99		0.99
Myeloablative	3 of 7 (42.9)		2 of 7 (28.6)	
Reduced intensity	12 of 25 (48)		8 of 25 (32)	
Stem cell source		0.23		0.53
PBSC	15 of 29 (51.7)		10 of 29 (34.5)	
UCB	0 of 3 (0)		0 of 3 (0)	
D/R relationship		0.24		0.41
Related	6 of 9 (66.7)		4 of 9 (44.4)	
Unrelated	9 of 23 (39.1)		6 of 23 (26.1)	
D/R HLA matching		0.70		0.42
HLA-matched*	10 of 22 (45.5)		6 of 22 (27.3)	
HLA-mismatched*	5 of 9 (55.6)		4 of 9 (44.4)	
D/R gender matching		0.65		0.29
Female donor for male recipient	3 of 5 (60)		3 of 5 (60)	
Others	12 of 27 (44.4)		7 of 27 (25.9)	
GVHD prophylaxis		0.53		0.53
CyA/tacro + MTX	3 of 6 (50)		3 of 6 (50)	
CyA/tacro + MMF	6 of 16 (37)		4 of 16 (25)	
Others	6 of 10 (60)		3 of 10 (30)	
Pre-transplant ATG		0.72		0.13
Yes	7 of 17 (41)		3 of 17 (18)	
No	8 of 15 (53)		7 of 15 (47)	
Time from alloHSCT to aGVHD diagnosis, median (range), days		0.17		0.25
<100d	8 of 21 (38.1)		5 of 21 (23.8)	
≥ 100d	7 of 11 (63.6)		5 of 11 (45.4)	
Overall grade aGVHD,		0.36		0.049
II	5 of 9 (55.6)		5 of 9 (55.6)	
III	8 of 15 (53.3)		5 of 15 (33.3)	
IV	2 of 8 (25)		0 of 8 (0)	
Organ involvement		0.46		0.99
Single organ	6 of 15 (40)		5 of 15 (33.3)	
Multiple organ	9 of 17 (52.9)		5 of 17 (29.4)	
Indication for MSC therapy		0.73		0.25
Steroid-refractory aGVHD	10 of 20 (50)		8 of 20 (40)	
Steroid-dependent aGVHD	5 of 12 (41.7)		2 of 12 (16.7)	
Time from grade II-IV aGVHD diagnosis to 1st MSC infusion, median (range), days		0.46		0.27
≤16	9 of 17 (52.9)		7 of 17 (41.2)	
>16	6 of 15 (40)		3 of 15 (20)	
1st MSC dose, *n*		0.036		0.049
1–2 $\times 10^6$ cells/kg	6 of 19 (31.6)		3 of 19 (15.8)	
3–4 $\times 10^6$ cells/kg	9 of 13 (69.2)		7 of 13 (53.8)	
2nd MSC infusion		0.99		0.99
No	11 of 24 (45.8)		8 of 24 (33.3)	
Yes	4 of 8 (50)		2 of 8 (25)	
